# Protective effect of berberine against LPS-induced endothelial cell injury via the JNK signaling pathway and autophagic mechanisms

**DOI:** 10.1080/21655979.2021.1915671

**Published:** 2021-04-25

**Authors:** Junping Guo, Wei Chen, Beibei Bao, Dayong Zhang, Jianping Pan, Mao Zhang

**Affiliations:** a Department of Emergency Medicine, Second Affiliated Hospital, Zhejiang University School of Medicine, Hangzhou, China; b Department of Clinical Medicine, Zhejiang University City College, School of Medicine, Hangzhou, China; c Cancer Institute of Integrated Traditional Chinese and Western Medicine, Zhejiang Academy of Traditional Chinese Medicine, Tongde Hospital of Zhejiang Province, Hangzhou, China; d Institue of Emergency Medicine, Zhejiang University, Hangzhou, China

**Keywords:** Berberine, endothelial cells, LPS, JNK inhibitor, autophagy

## Abstract

The role of autophagic mechanisms in the protective effect of berberine (BBR) on lipopolysaccharide (LPS)-induced injury in the endothelial cells human umbilical vein endothelial cells (HUVECs) and human pulmonary microvascular endothelial cells (HPMECs) was investigated. Cell viability, proliferation, and apoptosis were detected by the CCK-8 assay, the EdU kit, and flow cytometry, respectively, and autophagy-related protein expression, the number of autophagic vacuoles, and LC3 double-fluorescence were examined using western blot analysis, transmission electron microscopy, and confocal microscopy, respectively. LPS resulted in a decrease in the cell viability and proliferation of HUVECs and HPMECs and an increase in the number of apoptotic cells, while BBR treatment resulted in an increase in cell viability and proliferation, as well as a decrease in cell apoptosis. Furthermore, BBR could inhibit LPS-induced autophagy, as demonstrated by its inhibitory effects on the LC3-II/LC3-I ratio and Beclin-1 levels and its promotive effect on p62 expression. Addition of the autophagy inducer rapamycin (RAPA) aggravated LPS-induced injury, while treatment with the autophagy blocker 3-methyladenine (3-MA) attenuated the injury. Further, the protective effect of BBR was inhibited by rapamycin. JNK inhibition by SP600125 inhibited LPS-induced autophagy, and BBR could not alter the LPS-induced autophagy in HUVECs and HPMECs that were pretreated with SP600125. The present data indicate that BBR attenuated LPS-induced cell apoptosis by blocking JNK-mediated autophagy in HUVECs and HPMECs. Therefore, the JNK-mediated autophagy pathway could be a potential target for the prevention and treatment of cardiovascular disease.

## GRAPHICAL ABSTRACT

**Figure:**
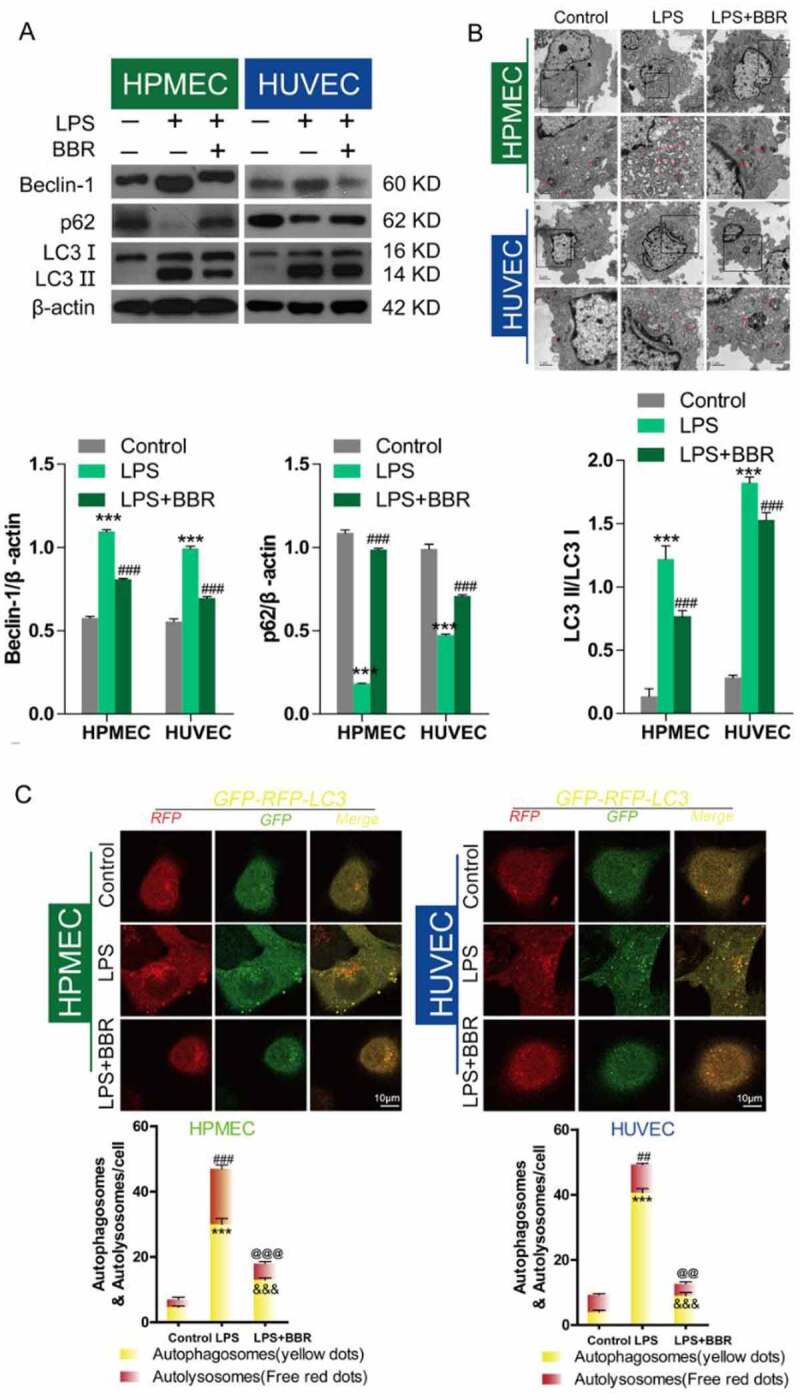

## Introduction

Cardiovascular diseases (CVDs), which have a high rate of mortality, are the leading cause of death worldwide [1–3]. Dysfunction of vascular endothelial cells (ECs) is an important feature of CVD [4–6]. Lipopolysaccharide (LPS) is an element of the outer membrane of all gram-negative bacteria, and it is also as a pro-inflammatory factor that directly acts on ECs to induce apoptosis and injury of the endothelium [7]. Therefore, it is important to investigate treatments that can reduce LPS-induced injury and loss to vascular ECs.

Berberine (BBR), a yellowish isoquinoline alkaloid, is the main active component of *Rhizoma coptidis*. It is a herbal medicine commonly used in traditional Chinese practice, and it has multiple potential uses in the treatment of inflammatory diseases such as diabetes mellitus, dyslipidemia, and liver fibrosis, via its antidiarrheal, antimicrobial, antipyretic, antiviral, and anti-inflammatory effects [8–11]. It also has preventive effects on many diseases, such as hypertension, arrhythmia, and congestive heart failure [12]. As a result of pharmacological advances in recent years, BBR has been used for the treatment of CVD [13]. In particular, BBR treatment has been shown to provide protection against EC apoptosis and injury induced by oxidized low-density lipoprotein via the cytochrome c-mediated caspase activation pathway [14]. Our previous research demonstrated that pretreatment with BBR could attenuate LPS-induced injury and reduce LPS-induced apoptosis in HUVECs via inhibition of JNK signaling [15]. In the present study, we sought to further explore the therapeutic potential and molecular targets of BBR with regard to its protective effects on human umbilical vein endothelial cells (HUVECs) and human pulmonary microvascular endothelial cells (HPMECs).

Autophagy, a lysosome-dependent catabolic process, which participates in the degradation and circulation of cytoplasmic organelles, is also very important for cell differentiation, homeostasis, and survival [16,17]. It is known that autophagy plays an important role in maintaining the structure and function of cardiovascular cells [18]. Autophagy is very important to CVD via regulation of EC functions [19]. BBR has a dual effect on autophagy, that is, inhibition and stimulation of autophagy [20]: It could attenuate apoptosis in Müller cells under high-glucose conditions through induction of autophagy and could also protect H9C2 cells from hypoxia-induced apoptosis through inhibition of autophagy [21,22].

Based on previous findings, we speculate that the protective effect of BBR on HUVECs and HPMECs involves autophagic mechanisms. Therefore, we will focus on investigating the role of autophagic mechanisms in the protective effects of BBR on LPS-induced HUVECs and HPMECs via inhibition of the JNK signaling pathway. Our study also provides evidence that autophagy plays a key role in the protective effect of BBR against LPS-induced HUVEC and HPMEC injury.

## Methods and material

### Cell culture

HUVECs were obtained from American Type Culture Collection (ATCC), and HPMECs were purchased from Shanghai Sixin Biotechnology Co. Ltd. All cells were maintained in RPMI1640 medium (GIBCO) containing 10% FBS in a humidified atmosphere containing 5% CO_2_ at 37°C.

### Cell viability assay

The CCK8 assay was used to examine cell viability. The cells were grown at a density of 5,000 cells/well in 96-well plates containing the 1640 medium (1% FBS) for 24 h. The cells were pretreated with 5 µM BBR for 24 h [15]. Next, 5 μg/ml LPS [15] was added for 24 h, and then rapamycin (RAPA, 100 nM) or 3-methyladenine (3-MA, 5 mM), as indicated, was added for 24 h. We added 10 μL of the CCK8 reagent to each well and incubated it for 3 h. Absorbance was read at 450 nm on a microplate reader (Dynex Technologies, Chantilly, USA) in order determine the optical density. Cell viability was standardized to that of the untreated controls.

### Western blot analysis

The cells were lysed with RIPA cell lysis reagent containing an inhibitor of proteinase and phosphatase (Sangon Biotech, Shanghai, China) at 4°C for 30 min. The protein concentrations were determined with the bicinchoninic acid protein assay reagent kit (Sigma, St. Louis, MO, USA). The samples (each containing 40 μg of protein) were isolated by 10% sodium dodecyl sulfate-polyacrylamide gel electrophoresis (SDS-PAGE) and transferred onto polyvinylidene fluoride membranes. The membranes were blocked with 5% nonfat milk containing Tris-buffered saline with 0.1% Tween 20 (TBST) for 2 h at 37°C, washed with TBST three times, and incubated with anti-Beclin-1, anti-p62, and anti-LC3 (diluted to 1:1000, Abcam) antibodies overnight. Following this, the membranes were washed again and incubated with the corresponding secondary antibodies for 1 h. The membrane was analyzed using enhanced chemiluminescence reagents in the dark, and the density of the bands was quantified using Image Lab 5.0.

### Cell proliferation analysis

Proliferation of the two EC lines was examined by a Click-iTEdU Imaging Kit according to the manufacturer’s protocol. Briefly, the cells were treated with BBR, LPS, RAPA, or 3-MA alone, or LPS combined with BBR, LPS combined with RAPA, LPS combined with 3-MA, respectively, and then 10 μM EdU for 2 h at 37°C. This was followed by fixation with 3.7% formaldehyde for 15 min and then permeabilization with 0.5% Triton X-100 for 20 min at room temperature. Cell nuclei were stained with Hoechst 33,342 (Invitrogen, USA) at a concentration of 5 μg/mL for 30 min and counted under the fluorescence microscope.

### Flow cytometry analysis

According to the manufacturer’s protocol, we used the Annexin V-FITC cell apoptosis detection kit to determine the number of apoptotic cells. Briefly, the ECs were treated with the indicated reagent (20 μg/ml LPS or 10 μM BBR) for 48 h. Following this, 2 × 10^5^ cells were collected to prepare a single-cell suspension. Then, the supernatant was discarded in the dark, and 5 μl/sample of FITC-labeled Annexin-V was added and incubated for 30 min. Following this, 5 μl/sample of PI was added and allowed to react for 5 min. The number of apoptotic cells was counted using flow cytometry.

### Confocal microscopy analysis

HUVECs and HPMECs were subjected to different treatments and then transfected with an mRFP-GFP-LC3 adenovirus. At post-transfection 48 h, the cells were fixed with 4% paraformaldehyde, and the expression of green (GFP) or red (mRFP) fluorescence was detected under a laser confocal fluorescence microscope. The yellow and red spots in the merged images represented autophagosomes and autolysosomes, respectively. The increase in the percentage of red spots in the merged image was considered to indicate autophagic flux.

### Transmission electron microscopy

The formation of autophagosomes was assessed using transmission electron microscopy (TEM) analysis. HUVECs and HPMECs were fixed in 2.5% glutaraldehyde overnight at 4°C, and then fixed in 1% buffered osmium tetroxide for 2 h. After dehydration with graded ethanol, they were embedded in epoxy resin and stained with uranyl acetate and lead citrate. TEM was used to obverse the stained sections.

### Statistical analysis

Experimental data were analyzed using Graphpad Prism 7.0 and expressed as the mean ± SD. Student’s *t*-test and one-way analysis of variance were performed to analyze differences between two independent groups and among multiple groups, respectively. P values of <0.05 were considered to indicate statistical significance.

## Results

### Effect of BBR on LPS-induced inhibition of cell viability and proliferation in HUVECs and HPMECs

**Figure 1.**
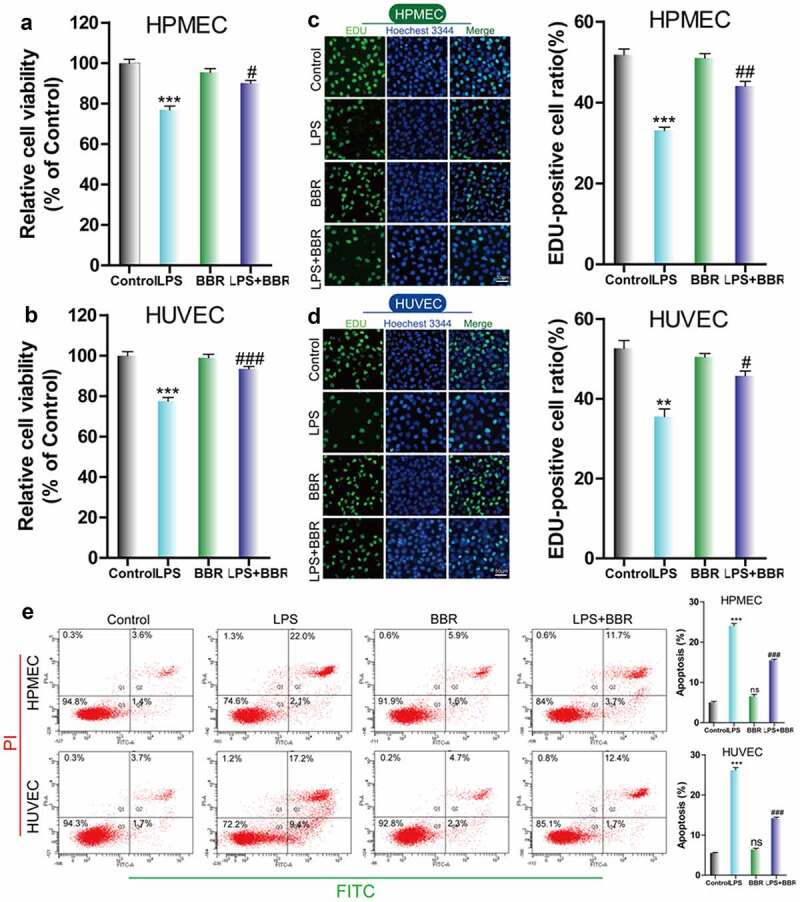
*Effect of BBR on the viability and proliferation of LPS-treated HUVECs and HPMECs*. A & B. Cell Counting Kit-8 (CCK-8) was used to analyze the effect of LPS on the cell viability of HUVECs and HPMECs, and the protective effect of BBR. ***P < 0.001 *vs*. control; #P < 0.05, ###P < 0.001 *vs*. LPS. C & D. EdU analysis of cell proliferation following LPS only, BBR only, and BBR+LPS treatment. All scale bars indicate 50 μm. **P < 0.01, ***P < 0.001 *vs*. the control; #P < 0.05, ##P < 0.01 *vs*. LPS. E. Apoptosis detection by flow cytometry analysis after treatment with LPS only, BBR only, and BBR+LPS. ***P < 0.001 *vs*. the control; ###P < 0.001 *vs*. LPS

To determine the protective effect of BBR on HUVECs and HPMECs that were injured by LPS treatment, we used CCK-8 and EdU to examine the cell growth of LPS-induced cells that were treated with BBR. We found that the viability and cell proliferation of HUVECs and HPMECs decreased after treatment with 5 μg/ml LPS, while the addition of 5 μM BBR resulted in an increase in cell viability and proliferation (Figure 1a–D). Furthermore, flow cytometry analysis showed that BBR could significantly reduce the increase in the number of HUVECs and HPMECs with LPS-induced apoptosis (Figure 1e). Thus, low-dose BBR can provide protection against LPS-induced inhibition of cell viability.

### Inhibitory effect of BBR on LPS-induced autophagy in HUVECs and HPMECs

**Figure 2.**
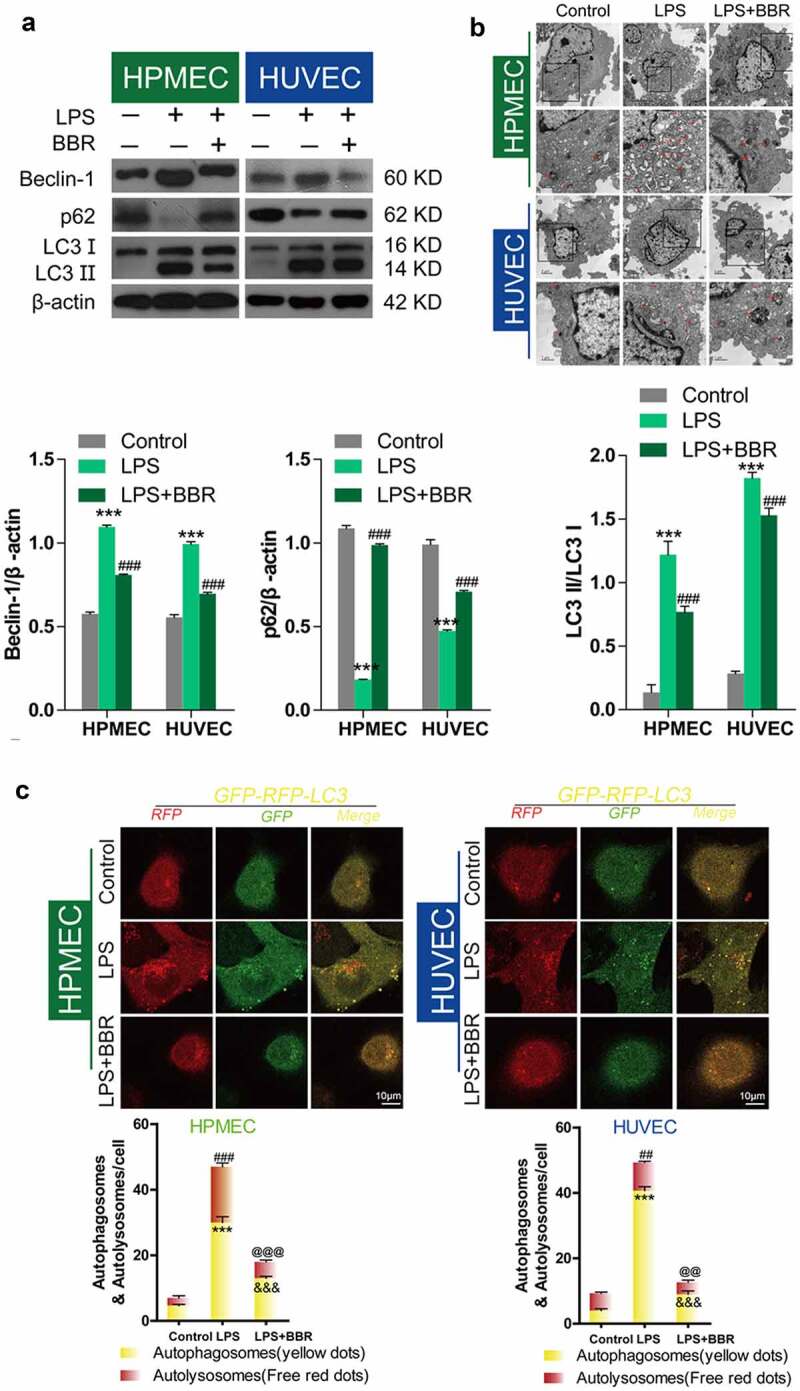
*Antagonistic effect of BBR on LPS-induced autophagy in HUVECs and HPMECs*. A. Beclin-1 and p62 expression and LC3-II accumulation after treatment with LPS alone, BBR alone, or BBR combined with LPS was determined by western blot analysis. B. Autophagic vacuoles were examined by transmission electron microscopy. C. LC3 double-fluorescence was determined by confocal microscopy analysis. Autophagosomes (yellow dots): ***P < 0.001 *vs*. the control; &&&P < 0.001 *vs*. LPS; Autolysosomes (free red dots): ##P < 0.01, ###P < 0.001 *vs*. the control; @@P < 0.01, @@@P < 0.001 *vs*. LPS

Western blot analysis showed that LPS treatment could upregulate Beclin-1 expression, downregulate p62 expression, and induce an increase in the LC3II/LC3I ratio, while BBR blocked these effects (Figure 2a), as reported in published articles. Transmission electron microscopy analysis showed that BBR could inhibit the increase in LPS-induced autolysosomes in HUVECs and HPMECs (Figure 2b). Confocal microscopy analysis with LC3 double-fluorescence staining showed that the number of both autophagosomes and autolysosomes was increased following treatment with LPS, while BBR blocked these effects of LPS in HUVECs and HPMECs (Figure 2c).

### Effect of RAPA and 3-MA on LPS-induced cell damage and autophagy in HUVECs and HPMECs

**Figure 3.**
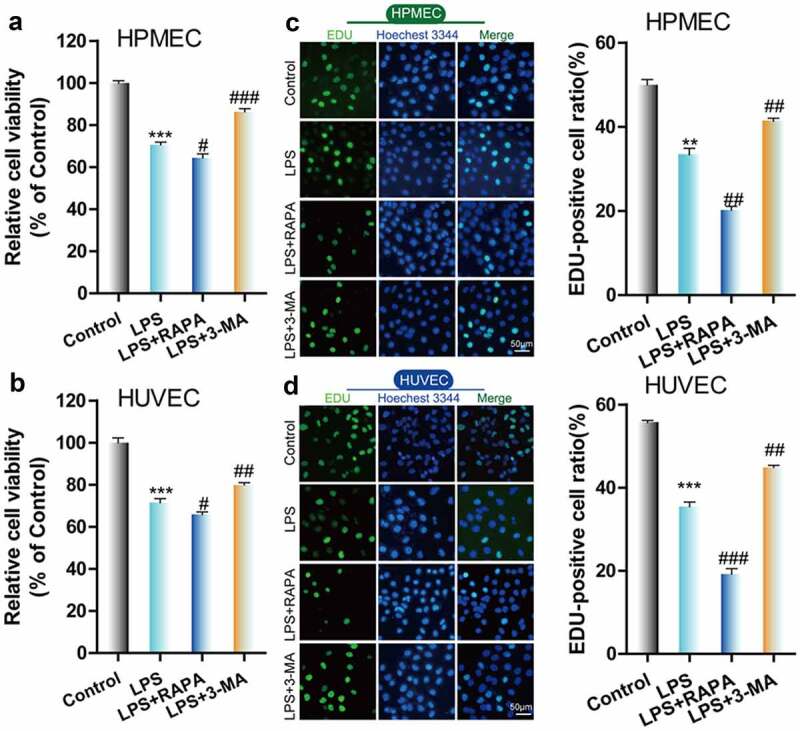
*Effect of RAPA and 3-MA on LPS-induced cell damage in HUVECs and HPMECs*. A & B. CCK-8 analysis of cell viability with or without RAPA or 3-MA treatment after LPS induction. ***P < 0.001 *vs*. the control; #P < 0.05, ##P < 0.01, ###P < 0.001 *vs*. LPS. C & D. Cell proliferation analysis with EdU with or without RAPA or 3-MA following LPS treatment

**Figure 4.**
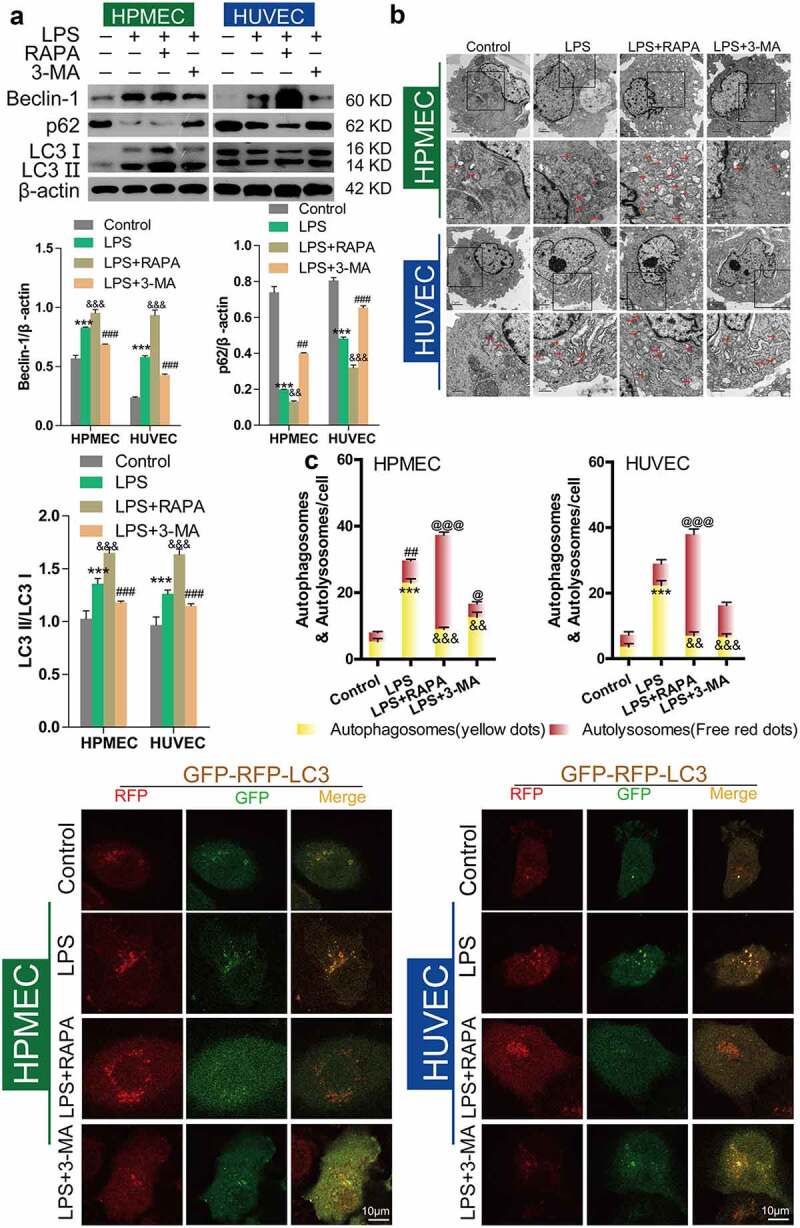
*Effect of RAPA and 3-MA on LPS-induced cell autophagy in HUVECs and HPMECs* A. Expression of autophagy markers (Beclin-1 and p62 expression and the LC3II/LC3I ratio) in different groups was determined by western blot analysis. B. Transmission electron microscopy analysis of the characteristic autophagic ultrastructures after different treatments. The arrows in the figures indicate autolysosomes. C. Confocal microscopy analysis of LC3 double fluorescence

Next, we used the autophagy inducer RAPA and the autophagy inhibitor 3-MA to verify the effect of autophagy on LPS-induced damage in HUVECs and HPMECs. The results indicated that the autophagy inducer RAPA caused a decrease in cell viability and proliferation following LPS induction, while the autophagy inhibitor 3-MA enhanced cell viability and proliferation, according to CCK-8 and EdU analysis (Figure 3a–D). Examination of the expression of the autophagy indicators by western blot analysis indicated that RAPA combined with LPS induced an increase in Beclin-1 and LC3II/LC3I and a decrease in p62 expression, while 3-MA combined with LPS caused a decrease in the level of the Beclin-1 protein and the LC3II/LC3I ratio and an increase in the expression of p62 (Figure 4a). TEM imaging of the characteristic autophagic ultrastructures indicated that RAPA induced an increase in the expression of autolysosomes, while 3-MA had the opposite effect in HUVECs and HPMECs (Figure 4b). Confocal microscopy analysis showed that compared with LPS treatment only, RAPA combined with LPS induced an increase in autophagy, while 3-MA combined with LPS caused the downregulation of autophagy (Figure 4c). These findings indicate that RAPA further aggravated LPS-induced damage, while treatment with 3-MA attenuated LPS-induced cell damage.

**P < 0.01, ***P < 0.001 *vs*. the control; ##P < 0.01, ###P < 0.001 *vs*. LPS.

***P < 0.001; ##P < 0.01 *vs*. the control; #P < 0.05, ##P < 0.01, @P < 0.05, @@@P < 0.001, &&P < 0.01, &&&P < 0.001 *vs*. LPS.

### Role of autophagy in the protective effect of BBR against LPS-induced HUVEC and HPMEC injury

**Figure 5.**
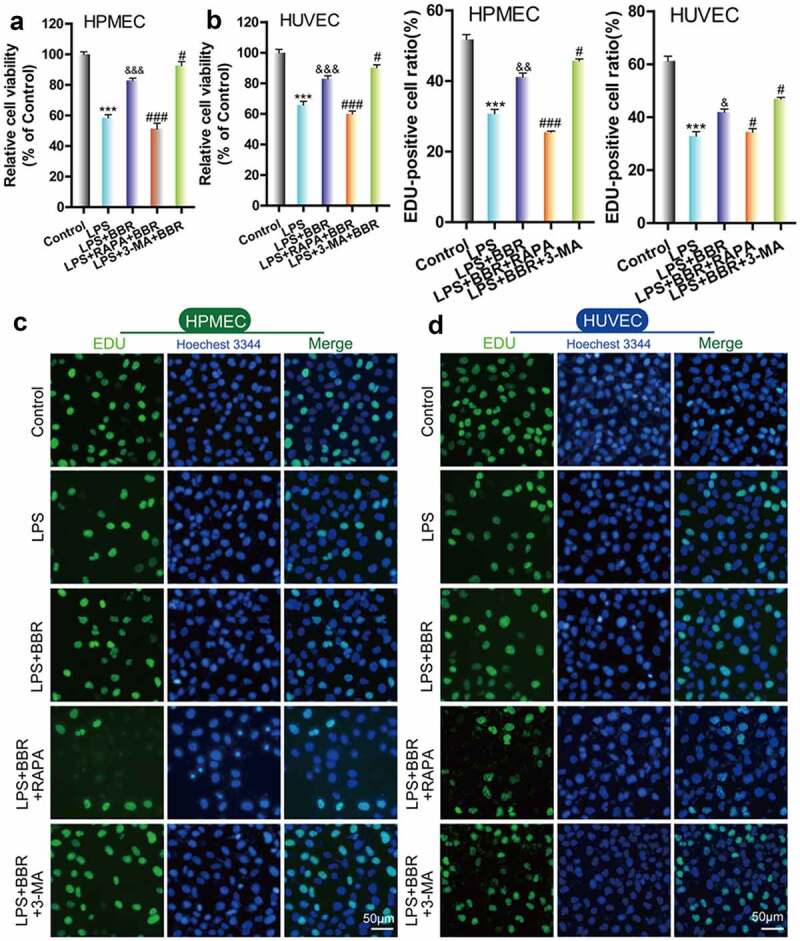
*Role of autophagy in BBR-mediated protection from LPS-induced cell injury in HUVECs and HPMECs*. A & B. CCK-8 verified the cell viability in the different treatment groups, such as BBR alone, BBR with RAPA, and BBR with 3-MA after LPS induction. ***P < 0.001 *vs*. the control; &&&P < 0.001 *vs*. LPS; #P < 0.05, ###P < 0.001 *vs*. LPS+BBR. C & D. EdU analysis of cell proliferation in HUVECs and HPMECs by BBR, BBR with RAPA, and BBR with 3-MA treatment after LPS induction

To confirm the role of autophagy in the protective effect of BBR on HUVECs and HPMECs, we combined BBR with the autophagy activator RAPA or inhibitor 3-MA. Cell activity and proliferation were further reduced and the cell damage was more serious after treatment with LPS and RAPA; this means that the protective effect of BBR could be reversed by RAPA. In contrast, treatment with LPS, 3-MA and BBR together enhanced cell activity and reduced cell damage (Figure 5 A–D).

***P < 0.001 *vs*. the control; #P < 0.05, ##P < 0.01 *vs*. LPS; &P < 0.05, &&&P < 0.001 *vs*. LPS.

### Inhibition of autophagy by BBR via inhibition of JNK

**Figure 6.**
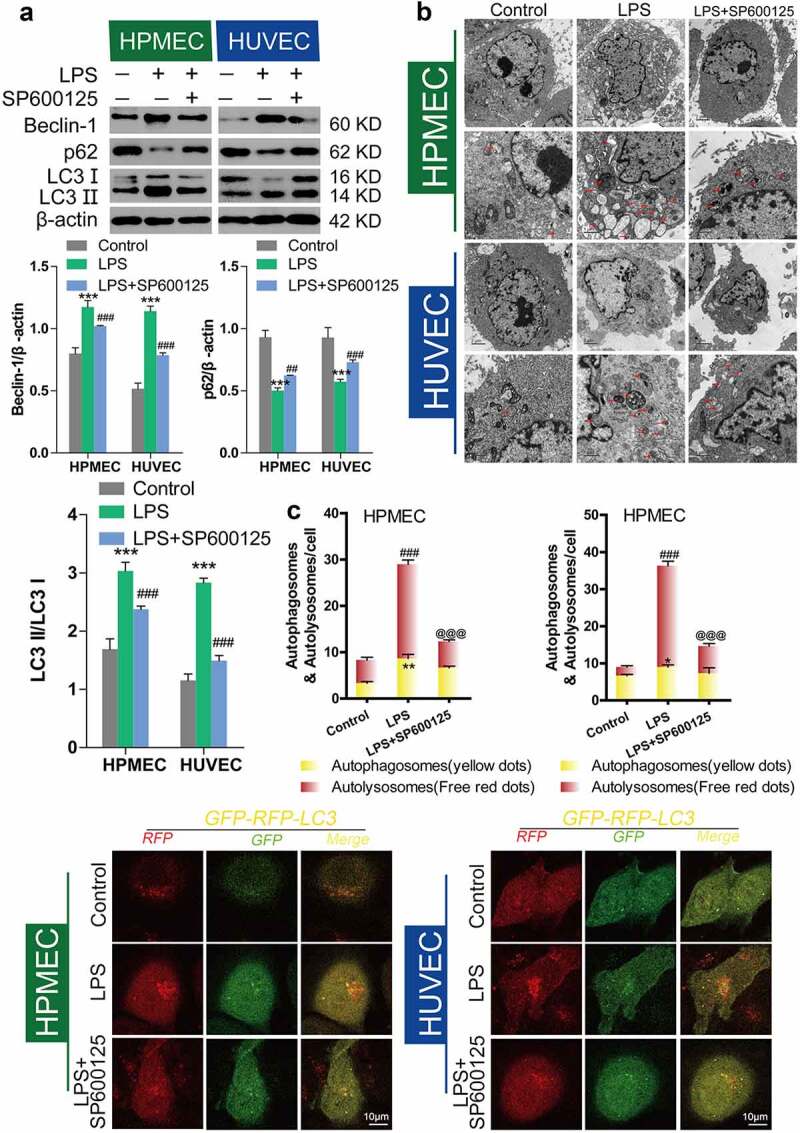
*Inhibition of LPS-induced autophagy in HUVECs and HPMECs treated with SP600125*. A. Changes in the expression of the autophagy indicators beclin-1, p62, and LC3 were examined by western blot analysis. B. In the transmission electron microscopy images, the ultrastructures observed are characteristic of autophagic cells and the arrows indicate autolysosomes. C. Confocal microscopy analysis of LC3 double fluorescence. Autophagosomes (yellow dot): *P < 0.05, **P < 0.01 vs. the control; autolysosomes (free red dots): ###P < 0.001 *vs*. the control; @@@P < 0.001 *vs*. LPS

**Figure 7.**
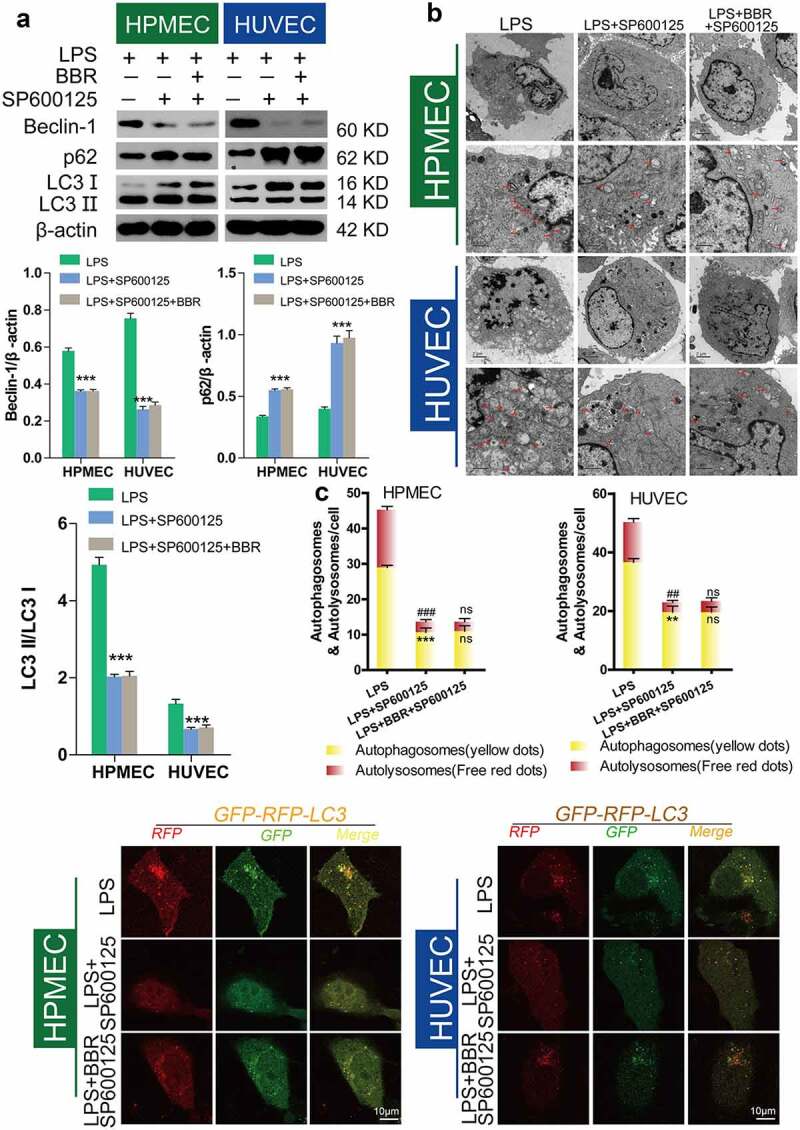
*Inhibition of autophagy in HUVECs and HPMECs by BBR via blocking of the JNK pathway* A. Changes in the expression of the autophagy indicators beclin-1, p62, and LC3 were determined by western blot analysis. B. Transmission electron microscopy images depict the ultrastructures of characteristic autophagic cells, and the arrows indicate autolysosomes. C. Confocal microscopy analysis of LC3 double fluorescence. Autophagosomes (yellow dots): **P < 0.01, ***P < 0.001 *vs*. LPS; autolysosomes (free red dots): ##P < 0.01, ###P < 0.001 *vs*. LPS

Previous research from our group confirmed that BBR could protect HUVECs and HPMECs from LPS-induced injury via inhibition of JNK signaling [15]. To elucidate the role of autophagic inhibition as a protective mechanism, we studied the effect of the JNK inhibitor SP600125 (40 nM) following LPS-induced autophagy. Compared with LPS-only treatment, SP600125 treatment led to downregulation of Beclin-1 and the LC3-II/LC3-I ratio and upregulation of p62 protein expression (Figure 6a). Transmission electron microscopy (TEM) observation of the number of autolysosomes showed that SP600125 could inhibit the increase in the number of autolysosomes induced by LPS treatment in HUVECs and HPMECs (Figure 6b). Confocal microscopy analysis with LC3 double-fluorescence staining showed that inhibition of JNK could block the occurrence of autophagy (Figure 6c). Furthermore, we determined the effect of adding BBR along with the JNK inhibitor. There were no statistically significant differences in Beclin-1 and p62 expression, or the LC-II/LC-I ratio, compared with the LPS group. This indicates that BBR could not block LPS-induced autophagy after JNK inhibition (Figure 7a). Transmission electron microscopy observation of the number of autolysosomes (Figure 7b) and confocal microscopy detection of LC3 double-fluorescence staining showed that after addition of the JNK inhibitor SP600125, BBR has no regulatory effect on autophagy. These findings indicate that BBR inhibits autophagy by inhibiting JNK (Figure 7c).

## Discussion

Our study proved that BBR blocked autophagy and apoptosis induced by LPS treatment in HUVECs and HPMECs. Moreover, we found that activation of the JNK pathway was very important in the process of autophagy, and the protective mechanism of BBR involved inhibition of JNK-mediated autophagy and apoptosis processes. These results verify that BBR has a protective effect on the initiation, progression, and mechanisms of CVD.

LPS can induce multiple endothelial responses as a proinflammatory molecule, and can also trigger EC injury [23]. A recent study has shown that LPS induces apoptosis in different types of ECs, such as HUVECs, HPMECs, and normal human microvascular ECs derived from the lung [24]. Here, we showed that the LPS-induced increase in cell apoptosis and decrease in cell viability and cell proliferation were significantly alleviated by BBR treatment of HUVECs and HPMECs. Thus, BBR decreases cell apoptosis and enhances cell viability and, therefore, proliferation in HUVECs and HPMECs [25]**.

Autophagy is a complicated process, and excessive autophagy and mild autophagy have dual functions in the regulation of cell apoptosis [26]. LPS-induced autophagy not only helps eliminate pathogens, but also alleviates toxin-induced cell damage. It has been reported that the protective effect of autophagy might be beneficial in cardiovascular disease, and BBR inhibited inflammation in macrophages by inducing autophagy [27]. Autophagy is strictly regulated by autophagy-related proteins and genes, and changes in LC3-II or the LC3-II/LC3-I ratio can reflect the activation of autophagic mechanisms [28,29]. LC3-I and LC3-II are present in the cytoplasm and within and outside of the autophagolysosome membrane, respectively [16]. Inhibition of autophagolysosome fusion, the degradation of autophagolysosome the inner membrane LC3-II can be prevented. Thus, a high ratio of LC3-II/LC3-I indicates that a large number of autophagosomes can be obtained via the conversion of LC3-I to LC3-II. We chose 3-MA and RAPA to confirm the effect of autophagy in LPS-induced HUVECs and HPMECs. 3-MA was found to increase cell viability and proliferation, decrease the levels of the autophagy markers Beclin-1 and LC3-II/LC3-I, and increase the levels of p62, while the autophagy inducer RAPA had the opposite effect. These studies indicate that autophagy might play a major role in regulating viability to protect ECs from LPS-induced injury.

JNK, a stress-activated protein kinase, plays key roles in apoptosis, cellular stress, and inflammation [30]. A previous study showed that SP600125 effectively inhibited LPS-induced injury in HUVECs and HPMECs by blocking the activation of the JNK pathway [15]. Additionally, research by Klein et al. has shown that JNK signaling may be involved in the regulation of autophagy [31]. This study further demonstrated that BBR could protect HUVECs and HPMECs from injury induced by LPS vis autophagy involving the JNK pathway. Consistent with the previous observations, our study confirmed that JNK inhibition with SP600125 blocked the effects of LPS, that is, the increase in Beclin-1 and the LC3-II/LC3-I ratio and the decrease in p62 expression, while BBR could not alter LPS-induced autophagy in HUVECs and HPMECs that were pretreated with SP600125. These findings imply that BBR promoted cell viability and proliferation and inhibited cell apoptosis and autophagy in LPS-stimulated HUVECs and HPMECs by activating the JNK signaling pathway.

## Conclusion

In conclusion, this study revealed that BBR could attenuate LPS-induced HUVEC and HPMEC apoptosis and autophagy via a mechanism that is potentially related to the inhibition of autophagy and JNK signaling.

## Supplementary Material

Supplemental Material

## Highlights

1. The effect of BBR on cell viability and proliferation by LPS-induction in HUVEC and HPMEC

2. BBR blocked LPS-induced autophagy in HUVEC and HPMEC cells

3. Role of RAPA and 3-MA in LPS-induced cell damage and autophagy in HUVEC and HPMEC cells

4. The role of autophagy in the protection of BBR against LPS-induced HUVEC and HPMEC injury
