# Supplementary figures and images for: Protective effect of berberine against LPS-induced endothelial cell injury via the JNK signaling pathway and autophagic mechanisms
Source: Bioengineered. 2021 Apr 25;12(1):1324–37. doi: 10.1080/21655979.2021.1915671 (PMC8806223; doi:10.1080/21655979.2021.1915671)

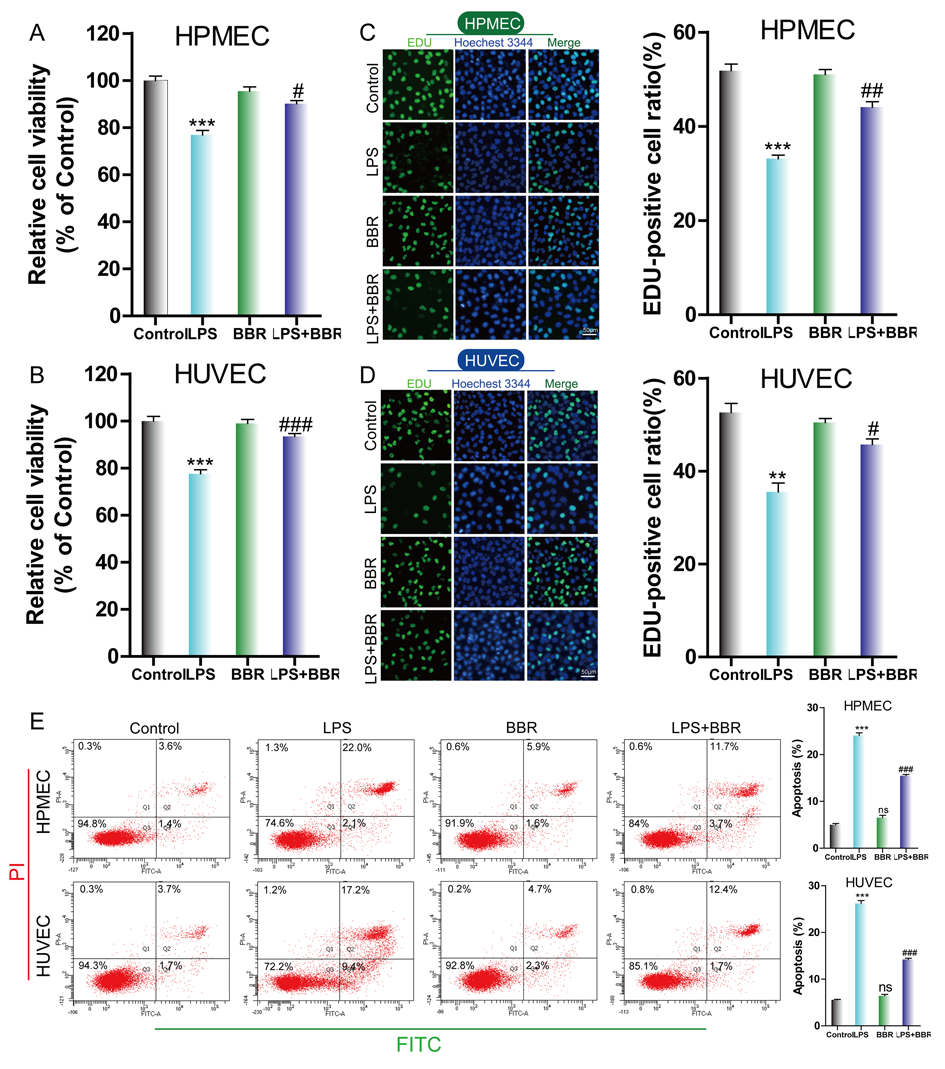

Supplement: Supplemental Material [file KBIE_A_1915671_SM8910.zip › Supplementary Figure 1.tif]
